# Multiple sclerosis and personality traits: associations with depression and anxiety

**DOI:** 10.1186/s40001-024-01772-0

**Published:** 2024-03-12

**Authors:** Saeed Vaheb, Yousef Mokary, Mohammad Yazdan Panah, Aysa Shaygannejad, Alireza Afshari-Safavi, Majid Ghasemi, Vahid Shaygannejad, Elham Moases Ghaffary, Omid Mirmosayyeb

**Affiliations:** 1 Isfahan Neurosciences Research Center, Isfahan University of Medical Sciences, Isfahan, Iran; 2 Students Research Committee, Shahrekord University of Medical Sciences, Shahrekord, Iran; 3 Department of Biostatistics and Epidemiology, Faculty of Health, North Khorasan University of Medical Sciences, Bojnurd, Iran; 4 Department of Neurology, School of Medicine, Isfahan University of Medical Sciences, Isfahan, Iran

**Keywords:** Personality traits, Depression, Anxiety, Multiple Sclerosis

## Abstract

**Background:**

Depression and anxiety are commonly observed in people with multiple sclerosis (pwMS). There is a growing body of literature supporting the hypothesis that personality traits can influence the mood disorders. This study aimed to investigate the personality traits and their relationships with depression and anxiety among pwMS.

**Methods:**

234 pwMS were involved in this cross-sectional study. Personality traits, depression, and anxiety were assessed using the NEO Five-Factor Inventory (NEO-FFI) and Hospital Anxiety and Depression Scale (HADS), respectively. Pearson's correlation coefficient and generalized linear model were employed to evaluate the relationships between demographic and clinical characteristics, NEO-FFI, and HADS subscales.

**Results:**

In pwMS, longer disease duration was significantly associated with lower level of conscientiousness (β = − 0.23, p = 0.008) and agreeableness (β = − 0.2, p = 0.01). Moreover, higher expanded disability status scale (EDSS) of pwMS had a significant relationship with higher level of neuroticism (β = 0.89, p = 0.01). Increased level of neuroticism was significantly correlated with lower level of extraversion (r = − 0.28, p < 0.001), openness (r = − 0.37, p < 0.001), agreeableness (r = − 0.31, p < 0.001), and conscientiousness (r = − 0.45, p < 0.001). PwMS with higher level of conscientiousness showed more extraversion (r = 0.23, p < 0.001), openness (r = 0.61, p < 0.001), and agreeableness (r = 0.41, p < 0.001). Elevated level of neuroticism was significantly associated with higher level of anxiety (β = 0.47, p < 0.001) and depression (β = 0.11, p < 0.001) among pwMS.

**Conclusion:**

The co-occurrence of depression and anxiety is probably associated with neuroticism among pwMS. Additionally, the impact of personality traits extends to influencing key disease aspects such as physical disability and disease duration in MS.

**Supplementary Information:**

The online version contains supplementary material available at 10.1186/s40001-024-01772-0.

## Introduction

Multiple sclerosis (MS) is an autoimmune disease of the central nervous system (CNS) characterized by demyelination.[1] Symptoms of MS can range from mild to severe and may be different from person to person. Cognitive impairment and changes in psychiatric function are some of the symptoms of MS. [2].

People with MS (pwMS) are more likely to experience symptoms of anxiety and depression as co-occurring conditions. [3] In approximately 30% of pwMS, symptoms of anxiety and depression are existed. [4, 5] In contrast, the prevalence of anxiety and depression in the general population was estimated to be 4% and 8.3%, respectively. [6, 7] There is also a tendency for pwMS to develop personality changes, such as increased irritability and apathy. Studies showed that 20% to 40% of pwMS exhibit personality changes. [2] The onset of neuropsychiatric disorders, such as depression and anxiety, may be triggered by uncommon personality traits in pwMS. [8].

Previous studies have examined the associations between anxiety, depression symptoms, and personality disorders in pwMS. However, the current literature has focused on newly diagnosed patients in far eastern cultures [9]. This limits the generalizability of these results. For example, some evidence suggested that personality disorders may be associated with prolonged disease duration or brain atrophy [10–12]. There are also cultural differences in the prevalence and occurrence of various personality and mood/anxiety disorders that need to be considered [13]. Hence, the current literature would benefit from investigations on patients in other cultures and with different disease durations.

Personality traits describe how an individual reacts to situations through thoughts, feelings, and behavior. [14] It consists of patterns of cognition, beliefs, and behaviors that are relatively stable. The Big Five model can explain many variations in these patterns, which has served as an effective theoretical framework. [15] These traits are framed within the Five Factor Model (FFM), encompassing five key dimensions: openness, conscientiousness, extraversion, agreeableness, and neuroticism. [10] Several studies have found that pwMS often exhibit dysfunctional personality traits related to neuroticism and conscientiousness. [16–18] This personality trait can adversely affect cognition, mood, and psychological well-being. [19] The precise mechanism linking personality disorders to the cognitive and mood states of pwMS remains incompletely understood despite validation through epidemiological studies. Additionally, research has proposed that personality traits might serve as moderators in the impact of brain atrophy on the cognitive and neuropsychiatric aspects of MS. [11, 20] Consequently, personality assessments should be incorporated as an integral part of the management of pwMS.

Based on the previous research, a comprehensive and large-scale study investigating personality disorders and their relationships with symptoms of anxiety and depression has not yet been conducted in Iran. This study aimed to investigate the personality traits and their relationships with symptoms of anxiety and depression levels among pwMS.

## Methods

This cross-sectional study was conducted on pwMS in Isfahan Kashani Hospital in Iran, between November 2022 and June 2023. The ethics committee approved this study at Isfahan University of Medical Sciences according to the Declaration of Helsinki and its later amendments (IR.ARI.MUI.REC.1401.230). All participants eligible for enrollment were thoroughly explained the study's aims and data collection methods and provided their informed consent to participate. Participants were informed that they had the right to refuse to participate in the survey at any time and asked that any personal information about them be removed from the database.

The inclusion criteria were as follows: (1) Age between 18 and 60 years (2) Confirmed diagnosis of MS by a neurologist [using McDonald’s diagnostic criteria [21]] (3) Consistently following the study conditions, e.g., completing the questionnaires and following the clinical interview (4) Disease duration of MS between 6 months to 10 years. Patients with other neurological or psychiatric disorders were excluded. The sample size was defined based on prior studies [10, 16, 22].

Patients meeting the inclusion criteria and undergoing routine neurological examinations at the clinic during the study period were included in this study upon completing the ethical consent form. The characteristics of the qualified patients were collected through interviews such as age, gender, employment status, educational status, disease duration, age at onset of disease, and expanded disability status scale (EDSS). Afterward, patients were asked to fill out the Persian version of the NEO Five-Factor Inventory (NEO-FFI) and Hospital Anxiety and Depression Scale (HADS) questionnaire to assess their personality traits and cognitive status [23, 24]. The NEO-FFI is a 60-item validated questionnaire that examines five personality traits: neuroticism, extroversion, openness, agreeableness, and conscientiousness. Each category consists of 12 questions; each level is evaluated on six criteria [24]. Concerning the evaluation of anxiety and depression, the HADS questionnaire includes 14 items and consists of two subscales: anxiety and depression. Each item is rated on a four-point scale, giving maximum scores of 21 for anxiety and depression. Scores of 11 or more on either subscale are considered significant cases of psychological morbidity, while scores of 8–10 represent borderline and 0–7 normal [23].

### Statistical analysis

The characteristics of the study population were described through frequency and percentage for qualitative variables, mean and standard deviation (SD) for quantitative variables with normal distribution, and median (interquartile) for quantitative variables with non-normal distribution. The relation between personality traits and demographic and clinical variables was checked through the generalized linear model. First, each variable was evaluated in a univariate model. Then, the significant variables in the previous step were entered into the final multivariate model. Pearson's correlation coefficients was applied to determine the correlations between personality traits. Statistical analysis was performed using SPSS Statistics for Windows, version 16. A significance level of 0.05 was considered.

## Result

### Preliminary analysis

A total of 234 pwMS were included in this study. The mean ± SD age of pwMS was 36.2 ± 9.7 years. The mean ± SD scores of depressions and anxiety were 10.6 ± 3.7 and 10.2 ± 3.9, respectively. More details of demographic, clinical, and psychological characteristics of pwMS are presented in Table 1.

**Table 1:** Demographic, clinical, and psychological data of pwMS. N = 234

Age; mean (SD)		36.25 (9.73)
Sex; n (%)	Female	197 (84.2)
	Male	37 (15.8)
Marital; n (%)	Single	59 (25.2)
	Married	165 (70.5)
	Divorced	9 (3.8)
	Widow	1 (0.4)
Education; n (%)	Non-academic	96 (41)
	Academic	138 (59)
Job; n (%)	Unemployed	143 (61.1)
	Employed	91 (38.9)
Duration of treatment; median (IQR)		2 (3)
Disease duration; median (IQR)		6 (8)
EDSS; median (IQR)		0 (1.5)
Neuroticism		31.82 (6.88)
Extraversion		26.60 (5.29)
Openness		24.63 (5.66)
Agreeableness		25.86 (6.35)
Conscientiousness		29.40 (7.09)
Anxiety HADS		10.27 (3.93)
Depression HADS		10.62 (3.75)

Data are presented as mean (SD). *HADS* hospital anxiety and depression Scale, *EDSS* expanded disability status scale, *PwMS* People with multiple sclerosis

### NEO-FFI and demographical and clinical characteristics of pwMS

Regarding neuroticism, the univariate model showed that married pwMS had a higher neuroticism score than singles (β = 2.503; p = 0.015). Longer disease duration (β = 0.221; p = 0.009) and higher EDSS (β = 1.130; p = 0.001) were associated with the increased level of neuroticism. However, after entering the significant variables in the multivariate model, the EDSS was the only variable that showed a significant relationship with depression (β = 0.890; p = 0.011) (Table 2).

**Table 2:** The generalized linear model between neuroticism and demographic and clinical characteristics of pwMS

Variables		Univariate		Multivariate	
		β (95% CI)	*p*-value	β (95% CI)	*p*-value
Age		0.009 (− 0.081,0.100)	0.844	–	–
Sex (Ref. = female)		− 1.483 (− 3.886,0.919)	0.226	–	–
Marital (Ref. = single)	Married	2.503 (0.494,4.512)	**0.015**	1.794 (− 0.206,3.795)	0.079
	Divorced	2.000 (− 2.740,6.740)	0.408	0.672 (− 4.018,5.362)	0.779
Education (Ref. = Non-academic)		− 0.418 (− 2.204,1.369)	0.647	–	–
Job (Ref. = Unemployed)		0.732 (− 1.069,2.534)	0.426	**–**	**–**
Duration of treatment		0.118 (− 0.207,0.444)	0.476	–	–
Disease duration		0.221 (0.055,0.388)	**0.009**	0.162 (− 0.005,0.329)	0.058
EDSS		1.130 (0.451,1.808)	**0.001**	0.890 (0.203,1.577)	**0.011**

*EDSS* expanded disability status scale, *PwMS* People with multiple sclerosis

Significant *p*-value are in bold

Extraversion was not associated with demographic and clinical characteristics of pwMS in univariate or multivariate models (p > 0.05) (Table 3).

**Table 3:** The generalized linear model between extraversion and demographic and clinical characteristics of pwMS

Variables		Univariate		Multivariate	
		β (95% CI)	*p*-value	β (95% CI)	*p*-value
Age		− 0.035 (− 0.104,0.035)	0.330	–	–
Sex (Ref. = female)		− 0.330 (− 2.185,1.524)	0.727	–	–
Marital (Ref. = single)	Married	− 0.857 (− 2.416,0.702)	0.281	–	–
	Divorced	1.976 (− 1.703,5.654)	0.292	–	–
Education (Ref. = Non-academic)		0.050 (− 1.325,1.426)	0.943	–	–
Job (Ref. = Unemployed)		− 0.105 (− 1.493,1.283)	0.882	**–**	**–**
Duration of treatment		0.175 (− 0.075,0.425)	0.169	–	–
Disease duration		0.002 (− 0.128,0.132)	0.977	–	–
EDSS		0.031 (− 0.502,0.565)	0.909	**–**	**–**

*EDSS* expanded disability status scale, *PwMS* People with Multiple sclerosis

In terms of openness, married pwMS scored lower than singles based on the univariate model (β = − 2.403; p = 0.004). PwMS with academic education had higher openness scores than patients with non-academic education (β = 1.719; p = 0.021). Only marital status showed a significant relationship with level of openness when the significant variables were entered into the multivariate model (β = − 2.068; p = 0.015) (Additional file 1: Table S1).

Concerning agreeableness, univariate and multivariate analyses revealed only a negative relationship between disease duration and level of agreeableness (β = − 0.203; p = 0.010 and β = − 0.203; p = 0.01, respectively) (Additional file 1: Table S2).

Also, the increase in disease duration was associated with the decreased  conscientiousness scores in univariate and multivariate analyses (β = − 0.232; p = 0.008 and β = − 0.232; p = 0.008, respectively) (Additional file 1: Table S3).

### HADS subscales and demographical and clinical characteristics of pwMS

According to a univariate model, married pwMS had higher anxiety scores than singles (β = 1.647; p = 0.005). Increased anxiety score was correlated with increased EDSS (β = 0.564; p = 0.004). The multivariate model also confirms these relationships (β = 1.410; p = 0.016 and β = 0.481; p = 0.016, respectively). The univariate and multivariate models showed that pwMS with higher level of neuroticism had higher level of anxiety, significantly (β = 0.48, p < 0.001, p = 0.472, p < 0.001, respectively). The univariate analysis found that anxiety had inverse associations with the levels of extraversion (β = − 0.251, p < 0.001), openness (β = − 0.182, p < 0.001), agreeableness (β = − 0.123, p = 0.002), and conscientiousness (β = − 0.213, p < 0.001) in pwMS. Multivariate analysis confirmed the inverse association between levels of extraversion and anxiety (β =− 0.072, p = 0.008), while it found a significant positive relationship between levels of agreeableness and anxiety (β = 0.055, p = 0.02) (Additional file 1: Table S4).

Regarding depression, the results of a univariate model showed that younger pwMS had higher neuroticism scores (β = − 0.059.; p = 0.018). A higher anxiety score was correlated with longer disease duration (β = 0.096; p = 0.04). Additionally, these relationships are confirmed by the multivariate model (β = − 0.082; p = 0.001 and β = 0.142; p = 0.003, respectively). The univariate and multivariate models revealed that pwMS with higher levels of neuroticism experienced significantly increased levels of depression (β = 0.214; p < 0.001 and β = 0.112; p < 0.001, respectively). The univariate analysis revealed that depression had an inverse relationship with levels of openness (β = − 0.181, p < 0.001), agreeableness (β = − 0.38, p < 0.001), and conscientiousness (β = − 0.168, p < 0.001) in pwMS. Using a multivariate analysis, it was confirmed that levels of depression and agreeableness were inversely related (β = − 0.333, p < 0.001) (Additional file 1: Table S5).

### Dimensional analyses

The Spearman correlation coefficients were calculated between the personality characteristics of pwMS, the level of depression, and the level of anxiety, as shown in Additional file 1: Table S6. Regarding personality traits, level of neuroticism had negative significant correlations with levels of extraversion (r = − 0.285, p < 0.001), openness (r = − 0.37, p < 0.001), agreeableness (r = − 0.319, p < 0.001), and conscientiousness (r = − 0.452, p < 0.001). The trait of extraversion showed positive significant correlations with levels of agreeableness (r = 0.143, p = 0.029) and conscientiousness (r = 0.237, p < 0.001). Regarding openness, it exhibited significant associations with level of agreeableness (r = 0.296, p < 0.001) and conscientiousness (r = 0.617, p < 0.001). The relationship between levels of agreeableness and conscientiousness was found to be substantial (r = 0.411, p < 0.001).

In relation to the HADS subscales, inverse correlations were observed between levels of anxiety and extraversion (r = − 0.338, p < 0.001), openness (r = − 0.263, p < 0.001), agreeableness (r = − 0.199, p < 0.05), and conscientiousness (r = − 0.385, p < 0.001). Additionally, level of anxiety showed a significant association with level of neuroticism (r = 0.84, p < 0.001). There were inverse relationships between level of depression and personality traits such as openness (r = − 0.272, p < 0.001), agreeableness (r = − 0.643, p < 0.001), and conscientiousness (r = − 0.318, p < 0.001). Neuroticism displayed a significant association with level of depression (r = 0.392, p < 0.001). Furthermore, a significant positive correlation was found between  levels of depression and anxiety (r = 0.392, p < 0.001) among pwMS.

## Discussion

Various aspects of pwMS’ mental state, including personality traits, levels of depression, and anxiety, were examined in this study. Additionally, we explored the factors contributing to the development of personality traits and mood disorders in pwMS. Our study aimed to shed light on the complex interplay between psychological factors and personality traits among pwMS. The exploration of personality traits in pwMS provides us with valuable insights into how pwMS deal with their condition and enables us to tailor their treatment strategies accordingly [25].

Our findings revealed that personality traits were associated with depression and anxiety. Depressed and anxious pwMS exhibited increased levels of neuroticism. In addition, pwMS exhibiting high levels of neurotic personality traits demonstrated reduced levels of agreeableness, openness, conscientiousness, and extraversion. Also, extraversion and openness are positively associated with agreeableness and conscientiousness. The personality traits were influenced by the clinical characteristics as well. PwMS with higher disability were more susceptible to experiencing heightened levels of neuroticism. Additionally, pwMS displaying higher conscientiousness and agreeableness had an extended disease duration compared to others.

Personality plays a significant role in pwMS, as individuals with this condition often experience frequent alterations in their personality [19]. Moreover, pwMS exhibit more dysfunctional personality traits, including lower levels of conscientiousness, extraversion, and agreeableness and higher levels of neuroticism. It has been proposed that the presence of personality dysfunctions in pwMS may be affected by other psychological disorders commonly seen in this population, specifically anxiety and depression [26].

According to previous studies, pwMS with mood disorders demonstrated elevated levels of neuroticism and reduced levels of conscientiousness compared to pwMS without mental disorders [13]. In the same way, pwMS who experience heightened indications of depression and anxiety have indicated higher levels of neuroticism and lower levels of extroversion, agreeableness, and conscientiousness when compared to the control group. It has been suggested that baseline personality traits such as neuroticism are risk factors for the subsequent development of major depression and anxiety in pwMS [16, 17]. High levels of neuroticism, which involve a heightened inclination towards experiencing negative emotions and psychological distress, constitute a significant risk factor for developing psychopathology, particularly depression and anxiety [27]. Certain mechanisms have elucidated the connection between personality traits and mood disorders.

A link between personality traits and excessive hypothalamic–pituitary–adrenal (HPA) axis activation has been established [28]. In this regard, higher neuroticism and lower openness and extraversion were associated with greater HPA activation and cortisol levels. It has been investigated that HPA dysregulation is involved in depressive and anxiety symptoms. Furthermore, this dysregulation of the HPA axis is a mediator between personality traits and the development of psychological disorders [29–32].

The interplay between psychological disorders and personality traits can be influenced by genetic components. The serotonergic system is a significant biological component involved in the development of depression. Neuroticism, associated with heightened anxiety, is linked to prolonged activation of the HPA axis, resulting in increased cortisol levels. This elevation may adversely affect the pathogenesis of MS, potentially elevating the risk of relapse [33]. In contrast, extraversion, marked by increased serotonin levels and emotional expression, tends to lower anxiety, reducing HPA axis activity and mitigating the risk of disease progression [12, 34].

There is a noteworthy link between the serotonin transporter protein promoter polymorphism (5HTT-LPR) genotype and major depressive disorder. The 5-HTT-LPR polymorphism located in the promoter region of the serotonin transporter (5-HTT) gene plays a role in controlling the transcription of the gene and the availability of 5-HTT at a physiological level [29]. The association between the binding of 5-HTT in the thalamus and the personality trait of neuroticism, as well as the facet of depression, was observed. Given that a high level of neuroticism is considered a risk factor for depression, it is possible that measuring 5-HTT binding in the thalamus could serve as a potential indicator of vulnerability to depression [35, 36]. Therefore, neuroticism mediates between the 5HTT-LPR genotype and major depression over a person's lifetime. This is in line with models of the causes of depression, which suggest that anxiety-related personality traits such as neuroticism play a significant role in increasing the chances of developing mood disorders [37, 38].

The correlations between personality traits and mood disorders could potentially be attributed to alterations in the structures of the brain. Previous studies have demonstrated a notable connection between brain atrophy and affective disorders in pwMS, particularly major depression [39]. Additionally, there is a remarkable relationship between decreased levels of extraversion, openness, and conscientiousness and a reduction in the volume of the cerebral cortex [40]. The brain network disruption and atrophy of frontal-parietal cortical regions are linked to low conscientiousness in MS [41]. The evidence indicates that cortical atrophy in MS has a negative effect on personality. Brain region degeneration would lead to the simultaneous presence of abnormal personality traits and mood disorders [10, 42].

Based on our findings, there was a link between depression and anxiety symptoms in pwMS. The presence of personality traits may act as a mediator in the relationship between depression and anxiety. The interaction between elevated levels of neuroticism and life stress can potentially trigger the onset of a depressive episode. Neuroticism could function as a predisposition to depression, leading to increased levels of persistent stress in one's life [43]. In addition, openness played a crucial role in linking initial stressful experiences to the development of depression [44].

Different disorders can be influenced by specific personality characteristics, such as neuroticism, which has the potential to impact an individual's health-related quality of life (HRQoL). In pwMS, personality characteristics can influence the disease progression in various ways, such as impacting the patient's readiness to pursue more risky treatment [45]. These personality traits can also influence the clinical characteristics associated with MS, including disability levels, physical and psychological functioning, disease duration, and treatment adherence. In this regard, our analysis indicated that patients with higher disability were more likely to show neuroticism. Regarding prior studies, it has been determined that disability and physical activity are linked to specific personality traits, particularly neuroticism and extraversion [26, 46, 47]. Furthermore, there is a consistent association between extraversion and both walking performance and muscle strength in adults. This connection may be explained by the fact that extraversion is linked to health-promoting behaviors, including engagement in physical activity [27]. However, we did not find any association between extraversion and disability. The connection between disability and personality traits can be explained by the impact of the association between treatment adherence and personality traits. As an example, pwMS with higher conscientiousness are less likely to experience disability worsening due to adhering to treatment.

Through observation that pwMS with specific patterns of personality traits experience changes in brain structures that affect the disease severity, it may be possible to explain the association between disability and personality traits. In addition, pwMS with lower levels of trait conscientiousness exhibit higher brain atrophy and impairment in white matter tracts connecting frontal, frontoparietal, and fronto-cingulate cortical regions (which involve interaction between action and reward) [48, 49].

As a result, personality traits can influence the clinical characteristics of patients, such as the level of disability. Patients who exhibit symptoms of depression and anxiety, along with high levels of neuroticism and openness, as well as low levels of conscientiousness, have been found to have difficulties in adhering to their treatment. Consequently, the lack of treatment adherence can negatively impact the duration and severity of their disability and disease [50]. Moreover, there is supporting evidence indicating that mood disorders are linked to the extent of disability in pwMS [25]. When considering the connection between personality traits and psychological factors, it can be inferred that there exists a correlation between disability and personality traits. Also, pwMS with prolonged disease duration exhibited lesser conscientiousness and agreeableness [41]. Conscientiousness is linked to improved markers of metabolic health, cardiovascular health, and inflammation. Moreover, conscientious individuals tend to engage in health-promoting behaviors, such as being more physically active and refraining from detrimental habits like consuming excessive amounts of alcohol and smoking. These behaviors contribute to reducing disease severity and duration [51].

According to mentioned evidence, personality traits significantly impact both the development of mood disorders and the adherence to treatment in pwMS. Accordingly, personality traits can influence the progression of the disease, including its severity, duration, and the level of disability experienced by pwMS.

A limited number of studies have implemented intervention strategies aimed at enhancing the treatment of personality disorders with the aim of improving the patients' HRQoL and minimizing their level of disability. In this specific context, Fuchs et al. employed a technology-aided approach, integrating in-person psychotherapy, telehealth psychotherapy, and a smartphone application designed as an external aid, specifically targeting pwMS with low conscientiousness. They found that pwMS who underwent this treatment experienced better work outcomes over three months compared to those without treatment [52]. Therefore, these interventions would be beneficial in assisting pwMS in effectively managing the challenges and limitations associated with their condition.

Personal affairs such as marriage would affect personality traits and mood disorders. According to our study, married patients showed increased anxiety symptoms and decreased openness. Marriage is regarded as a significant personal transformation that individuals undergo, often associated with changes in their personality. It is widely recognized as a key factor contributing to individual character alterations [53].

Our study highlights several strengths. Firstly, we examined the five major personality traits that notably influence the well-being and health of individuals with MS. Additionally, our study is strengthened by the use of a structured clinical psychiatric interview and validated assessments to evaluate personality traits, mood, and anxiety levels.

The present study comes with certain limitations. Firstly, it adopts a cross-sectional design, lacking prospective evidence, which makes establishing cause-and-effect relationships more challenging. Additionally, the use of the self-report HADS scale for assessing depression and anxiety introduces potential bias. Another drawback is the absence of a control group for comparison, leaving uncertainty about whether observed findings stem from normal personality variations or are inherent to neurogenic changes in MS. Lastly, for a more profound understanding, future research may explore the connection between mood/anxiety disorders and the perception of personality traits in individuals with MS, taking into account potentially limited self-awareness.

## Conclusion

In conclusion, this study revealed that depression and anxiety may associated with neuroticism among pwMS. Additionally, personality traits could affect the disease characteristics, such as physical disability and disease duration. The identification of personality disorders and their association with pathology of MS holds the potential for informing optimal treatment strategies, advancing insights into patient conditions, and facilitating the tailored provision of counselling and psychotherapy services. Further studies are required to clarify these relationships.

### Supplementary Information

**Additional file 1: Table S1.** The generalized linear model between openness and demographic and clinical characteristics of pwMS. **Table S2.** The generalized linear model between agreeableness and demographic and clinical characteristics of pwMS. **Table S3.** The generalized linear model between conscientiousness and demographic and clinical characteristics of pwMS. **Table S4.** The generalized linear model between anxiety and demographic and clinical characteristics of pwMS. **Table S5.** The generalized linear model between depression and demographic and clinical characteristics of pwMS. **Table S6.** Spearman correlations between personality traits and HADS subscales among pwMS.

## Data Availability

The data and materials supporting the findings of this study are available upon reasonable request. Researchers interested in accessing the data or materials should contact Dr. Vahid Shaygannejad at v.shaygannejad@gmail.com.
