# Supplementary material for: Multiple sclerosis and personality traits: associations with depression and anxiety
Source: Eur J Med Res. 2024 Mar 12;29:171. doi: 10.1186/s40001-024-01772-0 (PMC10929137; doi:10.1186/s40001-024-01772-0)
Supplement: Supplementary file 1 — Additional file 1: Table S1. The generalized linear model between openness and demographic and clinical characteristics of pwMS. Table S2. The generalized linear model between agreeableness and demographic and clinical characteristics of pwMS. Table S3. The generalized linear model between conscientiousness and demographic and clinical characteristics of pwMS. Table S4. The generalized linear model between anxiety and demographic and clinical characteristics of pwMS. Table S5. The generalized linear model between depression and demographic and clinical characteristics of pwMS. Table S6. Spearman correlations between personality traits and HADS subscales among pwMS. [file 40001_2024_1772_MOESM1_ESM.docx]

Table S1. The generalized linear model between openness and demographic and clinical characteristics of pwMS.

| Variables | | Univariate | | Multivariate | |
| --- | --- | --- | --- | --- | --- |
|  |  | β (95% CI) | *p*-value | β (95% CI) | *p*-value |
| Age |  | -0.023 (-0.098,0.051) | 0.538 | - | - |
| Sex (Ref.= female) |  | -0.329 (-2.312,1.654) | 0.745 | - | - |
| Marital (Ref.= single) | Married | **-2.403 (-4.044, -0.763)** | **0.004** | **-2.068 (-3.730, -0.407)** | **0.015** |
|  | Divorced | -2.151 (-6.021,1.719) | 0.276 | -1.848 (-5.699,2.002) | 0.347 |
| Education (Ref.= Non-academic) |  | **1.719 (0.264,3.173)** | **0.021** | 1.460 (-0.001,2.920) | 0.051 |
| Job (Ref.= Unemployed) |  | -0.560 (-2.043,0.922) | 0.459 | **-** | **-** |
| Duration of treatment |  | -0.170 (-0.438,0.097) | 0.211 | - | - |
| Disease duration |  | -0.132 (-0.270,0.006) | 0.062 | - | - |
| EDSS |  | -0.140 (-0.711,0.430) | 0.630 | **-** | **-** |

EDSS: Expanded Disability Status Scale, PwMS, People with Multiple Sclerosis.

Table S2. The generalized linear model between agreeableness and demographic and clinical characteristics of pwMS.

| Variables | | Univariate | | Multivariate | |
| --- | --- | --- | --- | --- | --- |
|  |  | β (95% CI) | p-value | β (95% CI) | p-value |
| Age |  | 0.040 (-0.044,0.123) | 0.351 | - | - |
| Sex (Ref.= female) |  | -1.438 (-3.657,0.781) | 0.204 | - | - |
| Marital (Ref.= single) | Married | -0.666 (-2.549,1.217) | 0.488 | - | - |
|  | Divorced | -0.450 (-4.892,3.992) | 0.843 | - | - |
| Education (Ref.= Non-academic) |  | 0.379 (-1.272,2.030) | 0.653 | - | - |
| Job (Ref.= Unemployed) |  | -1.262 (-2.920,0.397) | 0.136 | **-** | **-** |
| Duration of treatment |  | -0.233 (-0.533,0.066) | 0.127 | - | - |
| Disease duration |  | **-0.203 (-0.35, -0.049)** | **0.010** | **-0.203 (-0.35, -0.049)** | **0.010** |
| EDSS |  | -0.261 (-0.901,0.379) | 0.424 | **-** | **-** |

EDSS: Expanded Disability Status Scale, PwMS, People with Multiple Sclerosis.

Table S3. The generalized linear model between conscientiousness and demographic and clinical characteristics of pwMS.

| Variables | | Univariate | | Multivariate | |
| --- | --- | --- | --- | --- | --- |
|  |  | β (95% CI) | *p*-value | β (95% CI) | *p*-value |
| Age |  | -0.033 (-0.126,0.061) | 0.494 | - | - |
| Sex (Ref.= female) |  | 0.202 (-2.282,2.686) | 0.873 | - | - |
| Marital (Ref.= single) | Married | -0.566 (-2.658,1.525) | 0.596 | - | - |
|  | Divorced | -1.130 (-6.064,3.804) | 0.654 | - | - |
| Education (Ref.= Non-academic) |  | 0.815 (-1.025,2.655) | 0.385 | - | - |
| Job (Ref.= Unemployed) |  | -1.406 (-3.256,0.445) | 0.137 | **-** | **-** |
| Duration of treatment |  | -0.210 (-0.544,0.125) | 0.219 | - | - |
| Disease duration |  | **-0.232 (-0.404, -0.06)** | **0.008** | **-0.232 (-0.404, -0.06)** | **0.008** |
| EDSS |  | -0.132 (-0.847,0.582) | 0.717 | **-** | **-** |

EDSS: Expanded Disability Status Scale, PwMS, People with Multiple Sclerosis.

Table S4. The generalized linear model between anxiety and demographic and clinical characteristics of pwMS.

| Variables | | Univariate | | Multivariate | |
| --- | --- | --- | --- | --- | --- |
|  |  | β (95% CI) | p-value | β (95% CI) | p-value |
| Age |  | 0.002 (-0.050,0.053) | 0.946 | - | - |
| Sex (Ref.= female) |  | -0.962 (-2.332,0.409) | 0.169 | - | - |
| Marital (Ref.= single) | Married | **1.647 (0.503,2.792)** | **0.005** | **1.410 (0.262,2.557)** | **0.016** |
|  | Divorced | 1.154 (-1.546,3.855) | 0.402 | 0.800 (-1.883,3.483) | 0.559 |
| Education (Ref.= Non-academic) |  | -0.303 (-1.323,0.717) | 0.560 | - | - |
| Job (Ref.= Unemployed) |  | 0.423 (-0.606,1.451) | 0.421 | **-** | **-** |
| Duration of treatment |  | -0.004 (-0.190,0.182) | 0.968 | - | - |
| Disease duration |  | 0.089 (-0.007,0.185) | 0.069 | - | - |
| EDSS |  | **0.564 (0.175,0.954)** | **0.004** | **0.481 (0.090,0.872)** | **0.016** |
| Neuroticism |  | **0.480 (0.440,0.519)** | **<0.001** | **0.472 (0.426,0.518)** | **<0.001** |
| Extraversion |  | **-0.251 (-0.340,-0.161)** | **<0.001** | **-0.072 (-0.12,-0.019)** | **0.008** |
| Openness |  | **-0.182 (-0.268,-0.096)** | **<0.001** | 0.051 (-0.011,0.112) | 0.106 |
| Agreeableness |  | **-0.123 (-0.201,-0.046)** | **0.002** | **0.055 (0.009,0.100)** | **0.020** |
| Conscientiousness |  | **-0.213 (-0.279,-0.148)** | **<0.001** | -0.039 (-0.091,0.014) | 0.147 |

EDSS: Expanded Disability Status Scale, PwMS, People with Multiple Sclerosis.

Table S5. The generalized linear model between depression and demographic and clinical characteristics of pwMS.

| Variables | | Univariate | | Multivariate | |
| --- | --- | --- | --- | --- | --- |
|  |  | β (95% CI) | *p*-value | β (95% CI) | *p*-value |
| Age |  | **-0.059 (-0.108, -0.010)** | **0.018** | **-0.082 (-0.132, -0.031)** | **0.001** |
| Sex (Ref.= female) |  | -0.388 (-1.703,0.927) | 0.563 | - | - |
| Marital (Ref.= single) | Married | 0.261 (-0.850,1.372) | 0.645 | - | - |
|  | Divorced | 0.798 (-1.822,3.419) | 0.550 | - | - |
| Education (Ref.= Non-academic) |  | 0.369 (-0.606,1.344) | 0.458 | - | - |
| Job (Ref.= Unemployed) |  | 0.454 (-0.529,1.437) | 0.366 | **-** | **-** |
| Duration of treatment |  | 0.047 (-0.131,0.225) | 0.603 | - | - |
| Disease duration |  | **0.096 (0.005,0.188)** | **0.040** | **0.142 (0.048,0.236)** | **0.003** |
| EDSS |  | 0.266 (-0.111,0.643) | 0.166 | **-** | **-** |
| Neuroticism |  | **0.214 (0.150,0.278)** | **<0.001** | **0.112 (0.053,0.170)** | **<0.001** |
| Extraversion |  | -0.066 (-0.157,0.024) | 0.151 | **-** | **-** |
| Openness |  | **-0.181 (-0.262,-0.099)** | **<0.001** | -0.037 (-0.116,0.042) | 0.361 |
| Agreeableness |  | **-0.380 (-0.438,-0.322)** | **<0.001** | **-0.333 (-0.39,-0.271)** | **<0.001** |
| Conscientiousness |  | **-0.168 (-0.233,-0.104)** | **<0.001** | 0.022 (-0.046,0.091) | 0.519 |

EDSS: Expanded Disability Status Scale, PwMS, People with Multiple Sclerosis.

Table S6. Spearman correlations between personality traits and HADS subscales among pwMS.

|  | Neuroticism | Extraversion | Openness | Agreeableness | Conscientiousness | Anxiety | Depression |
| --- | --- | --- | --- | --- | --- | --- | --- |
|  | r (*p*-value) | r (*p*-value) | r (*p*-value) | r (*p*-value) | r (*p*-value) | r (*p*-value) | r (*p*-value) |
| Neuroticism | 1 |  |  |  |  |  |  |
| Extraversion | **-0.285 (<0.001)** | 1 |  |  |  |  |  |
| Openness | **-0.370 (<0.001)** | 0.041 (0.532) | 1 |  |  |  |  |
| Agreeableness | **-0.319 (<0.001)** | **0.143 (0.029)** | **0.296 (<0.001)** | 1 |  |  |  |
| Conscientiousness | **-0.452 (<0.001)** | **0.237 (<0.001)** | **0.617 (<0.001)** | **0.411 (<0.001)** | 1 |  |  |
| Anxiety | **0.840 (<0.001)** | **-0.338 (<0.001)** | **-0.263 (<0.001)** | **-0.199 (0.002)** | **-0.385 (<0.001)** | 1 |  |
| Depression | **0.392 (<0.001)** | -0.094 (0.154) | **-0.272 (<0.001)** | **-0.643 (<0.001)** | **-0.318 (<0.001)** | **0.352 (<0.001)** | 1 |

HADS: Hospital Anxiety and Depression Scale, PwMS, People with Multiple Sclerosis.
